# Insecticide resistance mapping in the vector of lymphatic filariasis, *Culex quinquefasciatus* Say from northern region of West Bengal, India

**DOI:** 10.1371/journal.pone.0217706

**Published:** 2019-05-29

**Authors:** Priyanka Rai, Minu Bharati, Abhisekh Subba, Dhiraj Saha

**Affiliations:** Insect Biochemistry and Molecular Biology Laboratory, Department of Zoology, University of North Bengal, West Bengal, India; Institute of Zoology Chinese Academy of Sciences, CHINA

## Abstract

*Culex quinquefasciatus* is a vector of lymphatic filariasis and vector control strategies normally involve the use of synthetic insecticides targeted against them. Extensive and uncontrolled use of these synthetic insecticides has led to the development of insecticide resistance in the mosquito vectors. In this context, to study the resistance status of *Cx*. *quinquefasciatus*, field populations were collected from three districts of Northern part of West Bengal and tested against insecticides (5% malathion, 0.05% deltamethrin, 0.05% lambdacyhalothrin,0.75% permethrin, 0.1% propoxur, 4% DDT and Temephos). Qualitative and quantitative enzyme assay was also conducted in order to find the role of detoxifying enzymes behind the development of insecticide resistance. This study revealed the presence of widespread resistance amongst the field populations of *Cx*. *quinquefasciatus* throughout the studied regions. Moreover, the result of native PAGE and biochemical enzyme assay may be linked to some extent in the involvement of the detoxifying enzymes in conferring resistance against insecticides in most of the tested *Cx*. *quinquefasciatus* populations. The present study involving the survey of resistance status may be of immense help during the implementation of vector control strategies throughout this region.

## Introduction

Vector-borne diseases are associated with major public health problems, mainly in the tropical and sub-tropical countries due to favourable climatic condition for the growth and proliferation of insect vectors and lack of proper sanitation. Among different insect vectors, mosquitoes are responsible for many dreaded tropical diseases like dengue fever, malaria, lymphatic filariais, west nile fever, Japanese encephalitis, chikungunya, yellow fever, zika, *etc* causing several million deaths and medical cases around the globe [1]. In the absence of proper treatment, some of these diseases may prove to be fatal while others might leave the patient with lifelong disability and impairment.

India due to its high relative humidity and lack of proper hygiene in some parts of the country is endemic to five mosquito-borne diseases *i*.*e*., dengue fever, Malaria, chikungunya, lymphatic filariasis and Japanese encephalitis [2]. The mosquito genus *Culex*has recorded 550 species and species like,*Culex quinquefasciatus*, *Cx*. *Vishnui*, *Cx*. *tritaenorhynchus*, *Cx*. *pseudovishnui*, *Cx*. *gelides and Cx*. *fuscocephala* are known to act as a vector for diseases like bancroftian filariasis, Japanese encephalitis, St Louis encephalitis virus and West Nile virus [3–5].

*Culex quinquefasciatus* Say in Southeast region of Asia is a vector for a filarial worm *Wuchereriabancrofti* that causes lymphatic filariasis [6]. In India, lymphatic filariasis or bancroftian filariasis is endemic in 20 states and 5 union territories thereby posing a threat of infection to about six hundred million people [7]. Mosquito-borne diseases in addition to having negative impact in the human health, hampers the socio-economic status of the affected people in a high rate and India being a developing country faces problems in combating these issues. Apart from this, the vector has also been a continuous biting nuisance mostly in and around areas near the mosquito breeding habitats. Lymphatic filariasis is in the list of one of the most important neglected tropical diseases, being the second leading cause of permanent and long-term disability in the world. India stands second after South Africa in the endemicity and transmission intensity of the disease [3].

Mosquitoes being vectors for a number of diseases have been a threat to the human society since last century. Though the severity of a disease depends upon the type of disease caused, control measures to avoid the spread and intensity of disease has always been one of the most important steps towards vector-borne disease control and management program. While there are many integrative preventive techniques that can be used for mosquito control, time and again the only effective way to control vector populations in disease endemic areas and decrease the densities of nuisance mosquitoes, is the application of synthetic chemical insecticides. Therefore, in order to control the vectors, chemical insecticides are extensively used against the vector population in the form of larvicides, indoor residual spraying (IRS), insecticides treated bed nets (ITNs) and long lasting insecticide treated bednets (LLITNs). Though there are mass chemotherapy programs for elimination of lymphatic filariasis at different regions of the country, yet vector control through the application of insecticides has still been the prime objective and strategy of filariasis control around the globe. Although insecticides play a central role in controlling vectors of diseases moreover, the unrestrained and unmanaged use of the chemical insecticides has resulted in the development of resistance in the mosquito population against these chemical insecticides in different corners of the world.

Insecticide resistance is defined as the ability of an insect to withstand the effects of an insecticide by becoming resistant to the toxic effects caused by them by means of adaptive natural selection and mutations [8]. Insects have developed resistance towards insecticides through four mechanisms *i*.*e*., behavioral modification, reduced penetration, metabolic detoxification and target-site insensitivity [9]. The latter two plays important role in the development of resistance and is widely studied. Reduced penetration of insecticides into the body is carried out by modifying the composition of the cuticle or increasing its thickness, mainly through enhanced deposition of structural components, such as epicuticular lipids and/or structural cuticular proteins [10]. This slow-down mechanism can delay insecticide molecules from reaching their protein targets thereby allowing detoxification enzymes more time to act and resulting in stronger resistant phenotypes. Development of insecticide resistance in mosquitoes occur mainly due to two major mechanisms i.e., target-site insensitivity and metabolic detoxification [11]. The former inhibits binding of the insecticides in the target site and the latter results in increased or modified activities of some detoxifying enzymes *i*.*e*., esterases, cytochromeP_450_ monooxygenases (CYP_450_) and glutathione S-transferases (GSTs).

Keeping in view the resistance development against insecticide in the vector population, the current study was conducted in *Culex quinquefasciatus* mosquitoes from total eight different sites in three districts of West Bengal to assess the insecticide susceptibility status and to find out the underlying biochemical mechanism of resistance development.

## Methodology

### Mosquito collection

**Fig 1:**
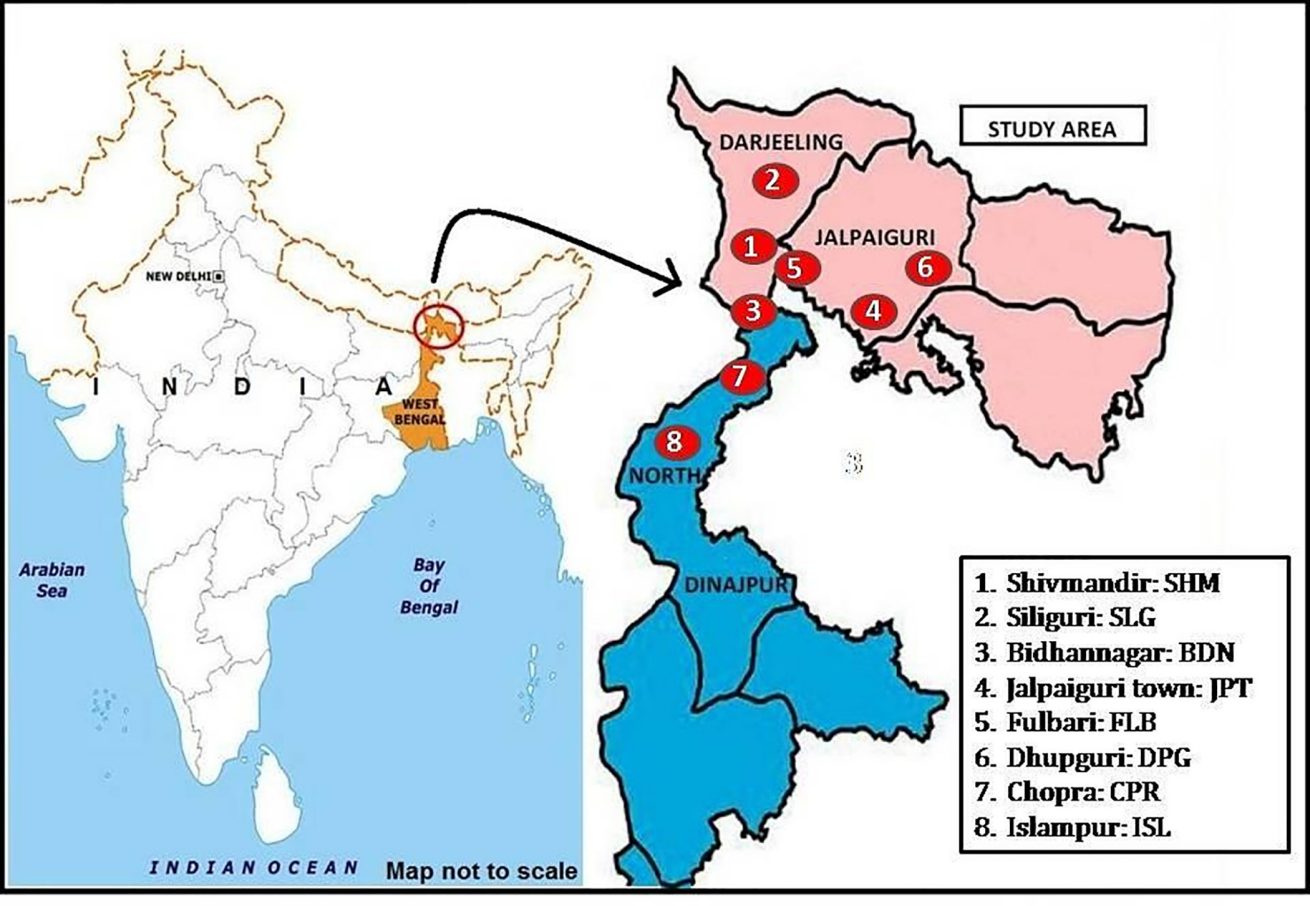
Map showing the sampling sites of *Culex quinquefasciatus* populations. Mosquitoes were collected from the natural breeding habitats from eight different sampling sites in three districts of northern region of West Bengal.

Field population of mosquitoes was collected in the month of April to November 2018 from eight different sites belonging tothree districts of sub-Himalayan region of West Bengal, India (Fig 1). Mosquito collection for the study was done from three densely populated areas of both Darjeeling district and Jalpaiguri district and two densely populated regions of Uttar Dinajpur district (S1 Table). Larvae and pupae were collected from various breeding habitats of *Culex quinquefasciatus* such as polluted drains, muddy pool containing stagnant water, plastic containers and cemented channels. Standard larval and adult identification keys of Tyagi *et al*., 2015 was followed to identify *Cx*. *quinquefasciatus* larvae and pupae [12]. Prior permission was taken from the land owner whenever a private land was examined. The collected mosquito population was reared to F1 generation in the laboratory under controlled temperature (25 ± 5°C) and relative humidity (70 ± 10%). F1 generation of each population was then used for the bioassay test and biochemical tests to maintain the homogeneity of the population.

### Rearing of susceptible population in the laboratory

*Cx*. *quinquefasciatus* larvae and pupae were collected from stagnant water bodies and drains in and around the university premises. In the laboratory, pupae were separated and kept in glass containers having sufficient amount of water to avoid over-crowding and larvae was kept in an enamel tray. A mosquito net was used to cover the entire setup. Powdered yeast and ground fish food was used as larval food and for the newly emerged adults, cotton balls soaked in 5% sugar solution were kept as food source. After the adults were 3–4 days old, blood food to the matured female mosquitoes was provided by placing a clean and trimmed rat in a small cage inside the rearing setup. Egg laying apparatus was set in a beaker containing tap water boiled with hay and then cooled down to room temperature. After 2–3 days the beaker was examined for egg rafts. Once egg rafts were found they were subsequently transferred to enamel trays containing tap water and ground fish food. After 1–2 days the eggs hatched into first instar larvae. The culture was carried on for subsequent generations and the tenth generation was used as the laboratory control/ susceptible population. The rearing was maintained under controlled temperature of 25 ± 2°C and relative humidity of 70–80%.

### Insecticide susceptibility tests

#### Insecticides

Temephos used for larval bioassay and impregnated papers of insecticides (0.05% Deltamethrin, 0.05% Lamdacyhalothrin, 0.75% Permethrin, 0.1% Propoxur, 5% Malathion and 4% DDT) used for adult bioassays were purchased from University Sains Malaysia.

#### Larval bioassay

Bioassay test against temephos was conducted as per the WHO guidelines [13]. Thirty late third instar to early fourth instar larvae of each population were exposed to two different doses of temephos *i*.*e*. 0.0200 ppm (WHO recommended dose) and 0.0125 ppm [National Vector-Borne Disease Control Programme recommended dose] in a 500mL glass beaker containing 249mL of dechlorinated tap water and 1 mL of the required concentration of temephos. Powdered yeast and ground fish food was provided as larval diet. Four other subsequent doses *i*.*e*. 0.0094, 0.00625, 0.0031, 0.0015 ppm were also prepared and used in order to determine the LC_50,_ LC_90_ and LC_99_ values of each population. A control was set up using dechlorinated tap water and every experiment had three replicates. Mortality percentage was calculated after 24 hours of exposure to larvicide.

#### Adult bioassay

WHO guidelines [14] were followed while performing the adult bioassay tests. Each insecticide impregnated papers were placed in susceptibility test vials into which 30non blood-fed 2–3 days old adult females from each population were introduced and exposed for an hour. The mosquitoes were then shifted to retention tube containing cotton ball soaked in 5% sucrose solution and maintained at laboratory conditions. Mortality percentage was recorded after 24 hours of exposure. Each experiment was performed in triplicates. In order to calculate the knock down time (KDT) of the synthetic pyrethroids and DDT counting of knocked down mosquitoes was done after every 10 minutes during one hour of insecticide exposure. A control was setup in which mosquitoes were exposed to acetone sprayed filter paper.

### Enzyme extraction

Individual non blood-fed adult *Cx*. *quinquefasciatus* were taken and homogenized in 200 μL of 0.02M ice cold sodium phosphate buffer (pH 7.2) with a teflon micro-pestle in a 1.5 mL centrifuge tube. The homogenate was centrifuged at 12000 rpm for 15 minutes in a centrifuge (eppendorf centrifuge r2418). The supernatant was stored at -20°C for further use in quantitative enzyme analysis.30 individual homogenate from each population was used for every assay.

#### Carboxylesterase activity assay

Minor modifications in the method of Van Asperen (1962) were used in a microplate reader to quantitate Carboxylesterases (CCEs) activity [15]. 20 μl enzyme source from each individual (n>30) was taken and mixed with 200 μl of working solution of α-napthyl acetate (α-NA) or β-napthyl acetate (β-NA). After 15 minutes 50μl of staining solution was added. The staining solution used was prepared by mixing two parts of 0.1% Fast Blue BB salt (FBBS) with five parts of 5% sodium dodecyl sulphate (SDS). Absorbance was recorded at 540 nm after 30 minutes of incubation period using microplate reader (Spectrostar nano, BMG labtech). Substrates used for α- Carboxylesterases and β- Carboxylesterases were α-napthyl acetate (α-NA) and β-napthyl acetate (β-NA) respectively. Standard curves of α-napthol and β-napthol were also prepared for the estimation of carboxylesterases activity. Blanks were set up using the same reaction mixture excluding the homogenate.

#### Monooxygenase activity assay

Cytochrome P_450_ (CYP_450_) activity was measured according to Brogdon *et al*., 1997 [16]. 20μl of the enzyme source was mixed with 200 μl of working solution of 3,3',5,5'-Tetramethyl benzidine (TMBZ) and 50μl3% H_2_O_2_ was used as a staining solution. Absorbance was recorded at 630nm using the microplate reader (Spectrostar nano, BMG labtech) after 2 hours of incubation. A standard curve for heme peroxidase activity was also prepared using cytochrome C horse heart type VI to quantitate the enzyme activity levels. Blanks were prepared using the reaction mixture without the enzyme homogenate.

#### Glutathione S-transferases (GSTs) activity assay

GSTs activity assay was carried out following the method of Habig, 1974 [17]. 3ml reaction mixture containing 0.05ml of 50mM CDNB (1-chloro-2, 4-dinitrobenzene), 0.15ml of reduced glutathione (GSH) was added to 2.79ml of 40mM potassium phosphate buffer (pH 7.2). After adding the homogenate, the mixture was kept in incubation for 2–3 minutes at 20°C and the increase in absorbance value was recorded for 5 minutes at 1 minute interval. The GST activity (***μ***M mg protein^-1^ min^-1^) was then calculated.

#### Protein quantification

Total protein content of the individual adult *Culex quinquefasciatus* was estimated using Folin-Lowry method [18] and absorbance taken at 630nm. Bovine serum albumin was used as a standard to obtain correct expression and specific activities of enzymes.

### Electrophoretic study of carboxylesterases

Native PAGE (Polyacrylamide gel electrophoresis) was carried on using equal amount of protein in tris-glycine (pH 8.3) at 120V for 4–5 hours at 4°C. 8% polyacrylamide gel was prepared to visualize the pattern of elevated esterase isozymes present in different populations. The gel was then stained according to Carvalho *et al*., 2003 [19]. The relative mobility (Rm) of various esterase isozyme bands were calculated based on mobility of esterases from anode to cathode using the formula individual band position ÷ *dyefont*. According to the Rm values, esterase isozymes were designated as EST I, II, and so on.

### Calculations

For the adult and larval bioassay, population showing mortality percentage of less than 90 is taken to be resistant population, above 98 as susceptible and mortality percentage between 90 and 98 is considered as having incipient resistance or resistance not confirmed [20]. Dose dependent mortality percentage was subjected to statistical analysis using probit in SPSS 21.0 software at 95% confidence interval in order to find out different LC_50_, LC_90_, LC_99_ values in ppm and KDT_50_ and KDT_99_ in minutes. The linear regression coefficient (r2) obtained was then used to calculate the linearity of experimental data. Resistance ratio (RR) was calculated by dividing the specific LC value of each wild population by that of SP, *i*.*e*. (RR90 = *LC*90 *of wildpopulation* ÷ *LC*99 *of SP*; RR50 = .*LC*50 *of wildpopulation* ÷ *LC*50 *of SP*). Twice the value of LC_99_ of each population was taken to be their respective diagnostic dose. In order to find out the association of all studied factors and insecticide resistance in *Culex quinquefasciatus* from the Northern districts of West Bengal, a Principal Component Analysis was performed in XLSTAT 2019.1 software.

## Results

### Larval bioassay

The result of larval bioassay against temephos in WHO recommended dose showed that all field populations were susceptible to temephos with mortality percentages ranging from 98.5–100 except SHM population from Darjeeling district with mortality percentage of 80 and therefore lying in resistance state (Table 1). Mortality of field population larvae in NVBDCP recommended dose varied as 4 of the tested populations *i*.*e*., BDN, JPT, FLB and CPR showed susceptibility towards temephos with cent percent mortality while DPG and ISL population showed intermediate resistance towards temephos with mortality percentage of 94.30 and 92.30 respectively and SLG population was resistant with 88.20% mortality rate. SHM population with only 44.23% mortality was found to be resistant towards NVBDCP *i*.*e*., Indian Government recommended dose of temephos.

**Table 1:** Larval bioassay of *Culex quinquefasciatus* against temephos.

Sites	WHO M (%) ± S.E	NVBDCP M (%) ± S.E	LC_50_ (ppm) ± S.E	LC_90_ (ppm)± S.E	LC_99_ (ppm) ±S.E	Recommended dose (ppm)	RR_50_	RR_90_
SHM	80.00 ± 0.06	44.23 ± 0.02	0.011 ± 6.0×10^−4^	0.027 ± 1.8×10^−3^	0.053 ± 2.6×10^−3^	0.106	11.00	18.00
SLG	100 ± 0.00	88.20 ± 0.04	0.001 ± 3.0×10^−4^	0.008 ± 1.3×10^−3^	0.035 ± 3.2×10^−3^	0.070	1.00	5.33
BDN	100 ± 0.00	100 ± 0.00	0.004 ± 3.7×10^−4^	0.007 ± 1.12×10^−3^	0.011 ± 3.37×10^−3^	0.022	4.00	4.67
JPT	100 ± 0.00	100 ± 0.00	0.001 ± 5.0×10^−4^	0.002 ± 3.0×10^−4^	0.004 ± 1.0×10^−3^	0.008	1.00	1.33
FLB	100 ± 0.00	100 ± 0.00	0.001 ± 1.25×10^−4^	0.004 ± 6.25×10^−4^	0.021 ± 2.5×10^−2^	0.042	1.00	2.67
DPG	98.5 ± 0.96	94.30 ±1.23	0.003 ± 2.25×10^−4^	0.008 ± 1.5×10^−3^	0.020 ± 8.0×10^−3^	0.040	3.00	5.33
CPR	100 ± 0.00	100 ± 0.00	0.002 ± 2.0×10^−4^	0.003 ± 1.25×10^−4^	0.005 ± 2.5×10^−4^	0.010	2.00	2.00
ISL	100 ± 0.00	92.30 ± 1.57	0.007 ± 5.0×10^−3^	0.017 ± 6.4×10^−3^	0.035 ± 1.4×10^−2^	0.070	7.00	11.33
SP	100 ± 0.00	100 ± 0.00	0.001 ± 4.0×10^−4^	0.0015 ± 9.0×10^−4^	0.002 ± 1.0×10^−3^	0.004	——	—-

LC_50_ values of the studied population ranged from 0.001–0.011 ppm with a maximum of 10 fold higher values when compared to LC_50_ value of SP (Table 1). Likewise, LC_90_ value ranged from 0.002–0.027 ppm and LC_99_ from 0.004–0.053 ppm. The site-specific recommended dose was higher for most of the studied population than the WHO recommended dose of temephos as an effective mosquito larvicide except for JPT and CPR (Table 1). RR_90_ values ranged from 1.33 in JPT population to 18 in SHM population. RR_90_ value <2 indicates susceptible population and population with RR_90_>2 is said to be resistant. Mortality percentage in the control setup was found to be below 5% therefore, no correction in calculation was needed.

### Adult bioassay

**Fig 2:**
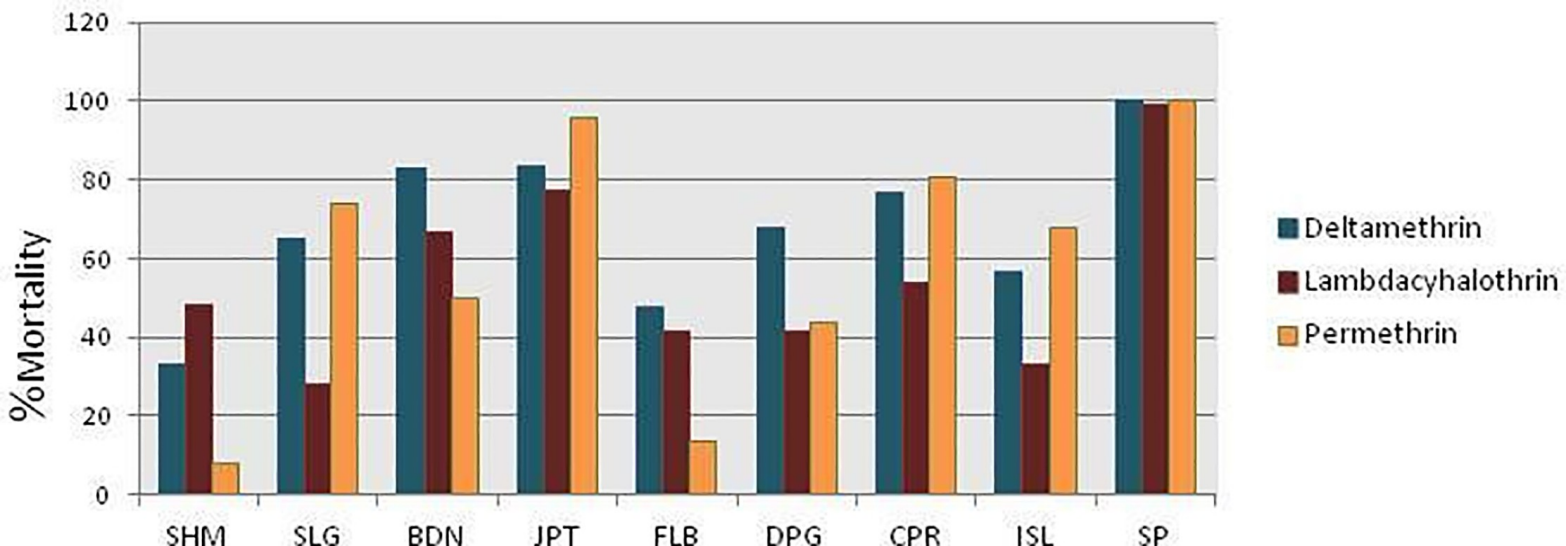
Chart depicting mortality rates against three synthetic pyrethroids (deltamethrin, lambdacyhalothrin and permethrin) in wild and SP of *Culex quinquefasciatus*.

**Fig 3:**
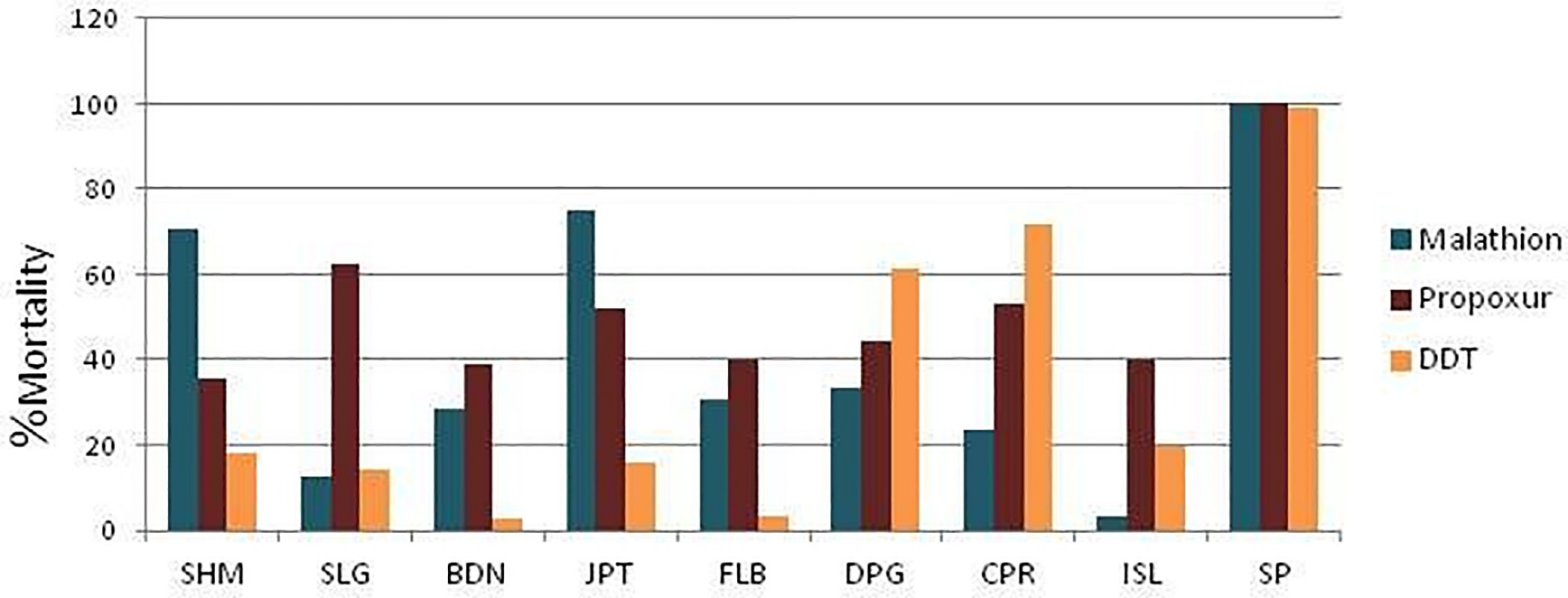
Chart depicting mortality rates against propoxur, malathion and DDT in wild and laboratory cultured populations of *Culex quiquefasciatus*.

Result of adult bioassay against six different insecticides is tabulated in (Table 2). Mortality percentage of all the studied populations against synthetic pyrethroids was less than 85% except for JPT (permethrin) (Fig 2). All of the studied populations showed resistance to Synthetic pyrethroids as evident by their lower mortality rates. Mortality percent of all of the studied populations against malathion, propuxur and DDT was less than 90 thereby revealing resistance against the same (Fig 3). KDT_50_ and KDT_99_ values of deltamethrin, lambdacyhalothrin, permethrin and DDT have been calculated and was found to be more than 60 minutes for all studied populations except SP.

**Table 2:** Susceptibility status of adult populations of *Culex quinquefasciatus* against six adulticides. (M–Mortality, n–total number).

sites	Deltamethrin	Lambdacyhalothrin	Permethrin	Malathion	Propoxur	DDT
	M (%) ± S.E	M (%) ± S.E	M (%) ± S.E	M (%) ± S.E	M (%) ± S.E	M (%) ± S.E
SHM	33.33 ± 0.85 (n = 98)	48.27 ± 1.35 (n = 96)	7.69 ± 0.65 (n = 93)	70.58 ± 0.82 (n = 100)	35.71 ± 0.07 (n = 97)	18.18 ± 0.64 (n = 94)
SLG	65.00 ± 1.62 (n = 102)	28.00 ± 0.84 (n = 93)	73.91 ± 1.24 (n = 98)	12.50 ± 1.34 (n = 90)	62.50 ± 0.84 (n = 96)	14.28 ± 0.36 (n = 96)
BDN	82.86 ± 1.07 (n = 95)	66.67 ± 0.86 (n = 102)	50.00 ± 0.51 (n = 90)	28.57 ± 0.68 (n = 96)	39.13 ± 1.52 (n = 94)	3.03 ± 0.81 (n = 99)
JPT	83.33 ± 0.58 (n = 99)	77.27 ± 0.23 (n = 91)	95.65 ± 0.46 (n = 101)	75.00 ± 1.37 (n = 92)	51.85± 0.92 (n = 102)	16.00 ± 1.08 (n = 99)
FLB	47.83 ± 1.07 (n = 94)	41.67 ± 1.09 (n = 90)	13.33 ± 0.91 (n = 99)	30.77 ± 1.12 (n = 100)	40.00 ± 1.01 (n = 93)	3.57 ± 0.54 (n = 94)
DPG	68.00 ± 0.61 (n = 104)	41.67 ± 0.46 (n = 95)	43.48 ± 1.08 (n = 94)	33.33 ± 0.61 (n = 104)	44.44 ± 0.63 (n = 98)	61.29 ± 0.67 (n = 103)
CPR	76.92 ± 0.84 (n = 96)	53.85 ± 0.63 (n = 95)	80.55 ± 1.02 (n = 93)	23.53 ± 0.52 (n = 99)	53.12 ± 0.76 (n = 105)	71.87 ± 0.72 (n = 100)
ISL	56.52 ± 0.98 (n = 90)	33.33 ±1.85 (n = 100)	67.78 ± 0.64 (n = 93)	3.22 ± 0.18 (n = 98)	40.00 ± 0.94 (n = 96)	20.00 ± 0.81 (n = 98)
SP	100 ± 0.00 (n = 92)	99.36 ± 0.26 (n = 90)	100 ± 0.00 (n = 90)	100 ± 0.00 (n = 91)	100 ± 0.00 (n = 93)	98.92 ± 0.09 (n = 90)

### Detoxifying enzymes’ activity assays

The quantitative activity of major detoxifying enzymes upon statistical analysis (p = 0.05) revealed varied level of expression in different populations (Table 3). α-CCEs activity ranged from a lowest of 0.759 mM mg protein^-1^ min^-1^ (in JPT) to a highest value of 5.948 mM mg protein^-1^ min^-1^ (in SLG) and β-CCEs activity was recorded to be least in CPR (0.640 mM mg protein^-1^ min^-1^) and highest in ISL (4.537 mM mg protein^-1^ min^-1^). The studied populations of mosquito showed 3.95–30 fold higher α-CCEs activity and 3.17–15.65 times higher β-CCEs activity when compared to the α-CCEs activity (0.192 mM mg protein^-1^ min^-1^) and β-CCEs activity (0.202 mM mg protein^-1^ min^-1^) of SP. CYP_450_ activity values ranged from 0.358 nM mg protein^-1^ min^-1^ to 2.135 nM mg protein^-1^ min^-1^ and showed a maximum of 34 times higher value than the CYP_450_ activity of SP (0.062 nM mg protein^-1^ min^-1^). Quantitative activity of GSTs was found to be 80 fold higher than SP (22.43×10^-6^mM mg protein^-1^ min^-1^) in SHM (18.12×10^-3^mM mg protein^-1^ min^-1^) that showed the highest GSTs activity among all of the studied field populations.

**Table 3:** Quantitative activity of major detoxifying enzymes in *Culex quinquefasciatus* populations.

sites	α-carboxyesterases activity (mM mg protein^-1^ min^-1^ ± S.E)	β-carboxyesterases activity (mM mg protein^-1^ min^-1^ ± S.E)	Monooxygenases activity (nM mg protein^-1^ ± S.E)	GSTs activity (mM mg protein^-1^ min^-1^ ± S.E)
SHM	5.948 ± 0.761^d^	3.428 ± 0.317^d^	1.523 ± 0.041^d^	18.12×10^−3^± 2.1×10^-3d^
SLG	4.088 ± 0.237^c^	2.857 ± 0.463^c^	0.929 ± 0.072^c^	15.41×10^−4^ ± 2.6×10^-4b^
BDN	1.914 ± 0.147^b^	2.580 ± 0.179^c^	2.135 ± 0.233^e^	31.72×10^−4^ ± 3.9×10^-4c^
JPT	0.759 ± 0.047^b^	0.713 ± 0.032^b^	0.358 ± 0.091^b^	76.68×10^−5^ ± 1.4×10^−4 b^
FLB	5.213 ± 0.361^d^	2.522 ± 0.145^c^	1.337 ± 0.338^d^	79.81×10^−5^± 3.6×10^-5b^
DPG	5.166 ± 0.410^d^	1.840 ± 0.067^c^	1.298 ± 0.145^d^	48.34×10^−4^ ± 1.8×10^-4c^
CPR	1.390 ± 0.040^b^	0.640 ± 0.030^b^	0.841 ± 0.083^c^	33.00×10^−4^ ± 6.4×10^-4c^
ISL	5.293 ± 0.123^d^	4.537 ± 0.401^d^	0.980 ± 0.082^c^	11.38×10^−3^ ± 2.9×10^-3d^
SP	0.192 ± 0.03^a^	0.202 ± 0.025^a^	0.062 ± 0.021^a^	22.43×10^−6^ ± 1.4×10^−6 a^

Within columns, means followed by the same letter do not differ significantly (P = 0.05) in Tukey’s multiple comparison test (HSDa)

### Native polyacrylamide gel electrophoresis

**Fig 4:**
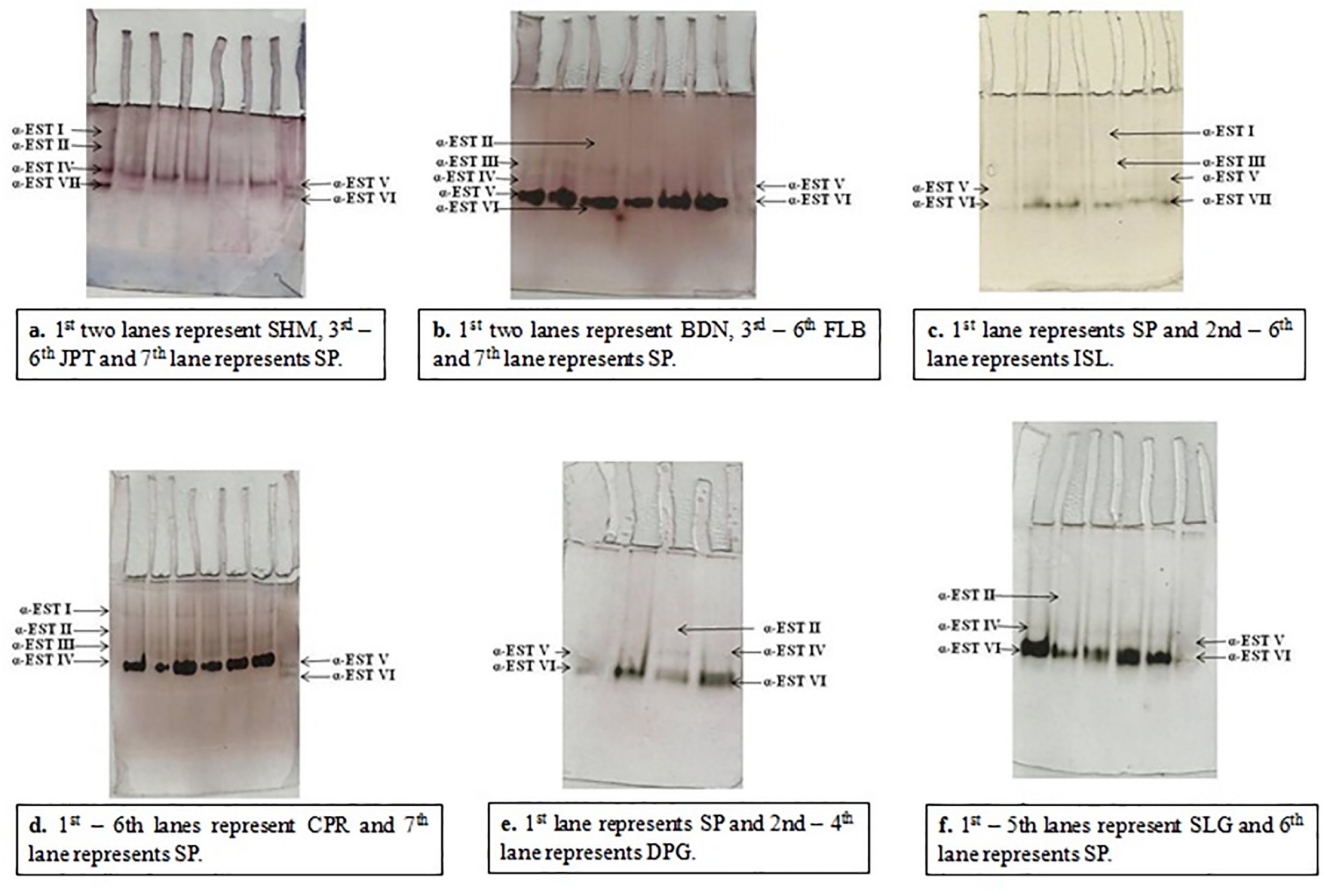
Qualitative analysis of α- carboxylesterases in *Culex quinquefasciatus* populations from three districts of West Bengal.

**Fig 5:**
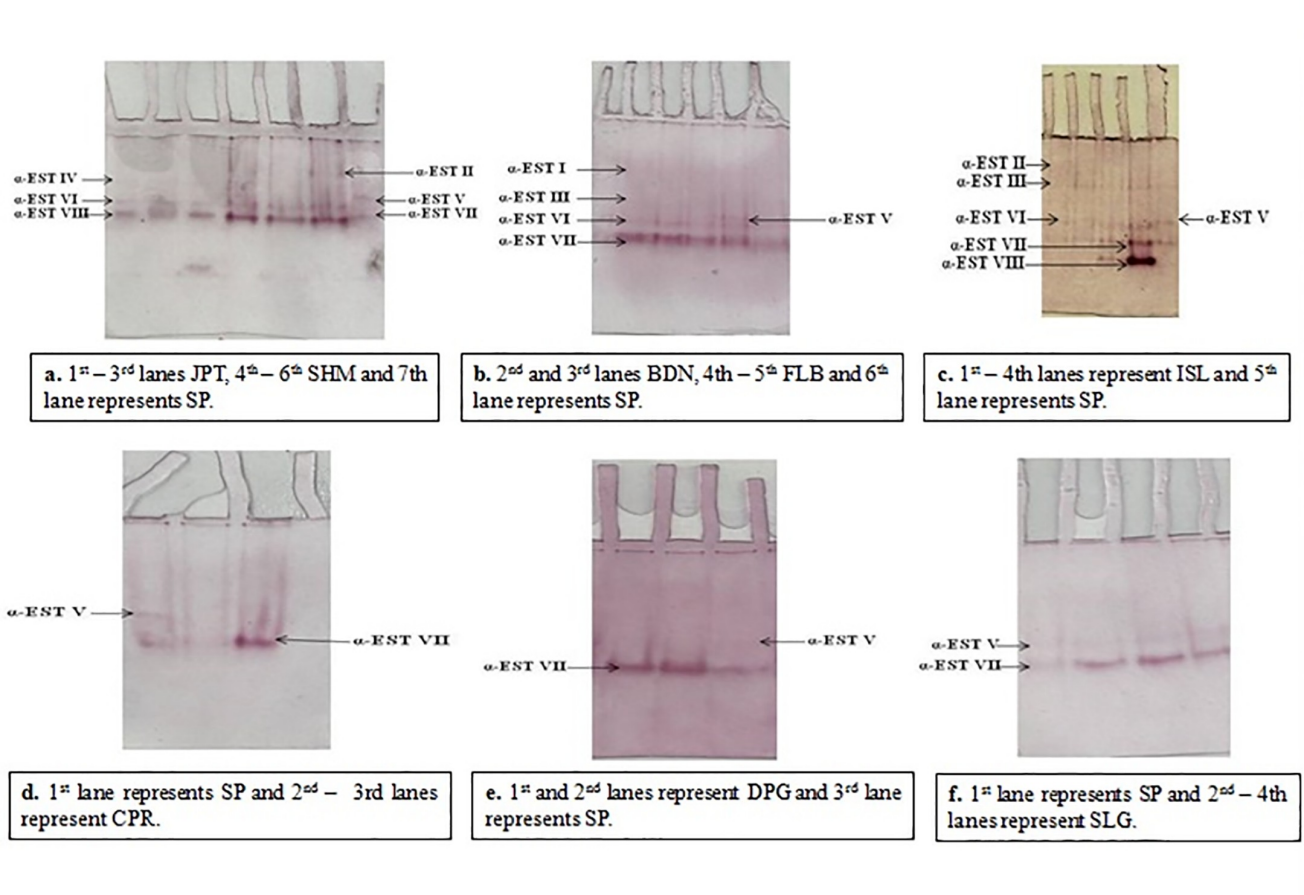
Qualitative analysis of β- carboxylesterases in *Culex quinquefasciatus* populations from three districts of West Bengal.

Based on the Rm of each band obtained in 8% polyacrylamide gel, the studied populations expressed a total of 7 different isozymes for α-esterases and 8 different bands for isozymes of β-esterases (Tables 4 and 5). The isozymes have been named subsequently as α-EST I, α-EST II, α-EST III, α-EST IV, α-EST V, α-EST VI, α-EST VII for α-esterases (Fig 4) and for the enzyme β-esterases as β-EST I, β-EST II, β-EST III, β-EST IV, β-EST V, β-EST VI, β-EST VII and β-EST VIII (Fig 5). None of the tested population expressed all the bands. Maximum number of α-esterases isozymes expressed was calculated to be 4 in an individual population while JPT showed a minimum of 2 bands. Moreover, a maximum of 5 different isozymes have been expressed for β-esterases in a single population whereas, CPR and DPG populations expressed a single band only (Table 5).However, the bands expressed for both α-esterases and β-esterases of all studied sites showed varying intensities (Tables 4 and 5).

**Table 4:** Different α-CCEs isozymes in *Culex quinquefasciatus* populations.

	SHM	SLG	BDN	JPT	FLB	DPG	CPR	ISL	SP
α-EST I	+	−	−	−	−	−	+	+	−
α-EST II	+	+	−	−	+	+	+	−	−
α-EST III	−	−	+	−	+	−	+	+	−
α-EST IV	++	++	+	−	+	+	+++	−	−
α-EST V	−	−	+++	+	−	−	−	++	+
α-EST VI	−	+++	−	−	+++	++	−	−	++
α-ESTVII	+++	−	−	++	−	−	−	++	−

+++ denotes high intensity

++ denotes moderate intensity

+ denotes low intensity and

–denotes absence of α-CCEs isozymes.

**Table 5:** Different β-CCEs isozymes in *Culex quinquefasciatus* populations.

	SHM	SLG	BDN	JPT	FLB	DPG	CPR	ISL	SP
β-EST I	−	−	+	−	+	−	−	−	−
β-EST II	++	−	−	−	−	−	−	+	−
β-EST III	−	−	+	−	+	−	−	+	−
β-EST IV	+	−	−	+	−	−	−	−	−
β-EST V	−	+	−	−	++	−	−	−	+
β-EST VI	−	−	++	+	++	−	−	+	−
β-EST VII	++	+++	+++	−	+++	++	+++	+++	+
β-EST VIII	+++	−	−	++	−	−	−	+++	−

+++ denotes high intensity

++ denotes moderate intensity

+ denotes low intensity and

–denotes absence of β-CCEs isozymes.

### Association between the observed insecticide resistance in *Cx*. *quinquefasciatus* and the quantitative activity of major detoxifying enzymes

**Fig 6:**
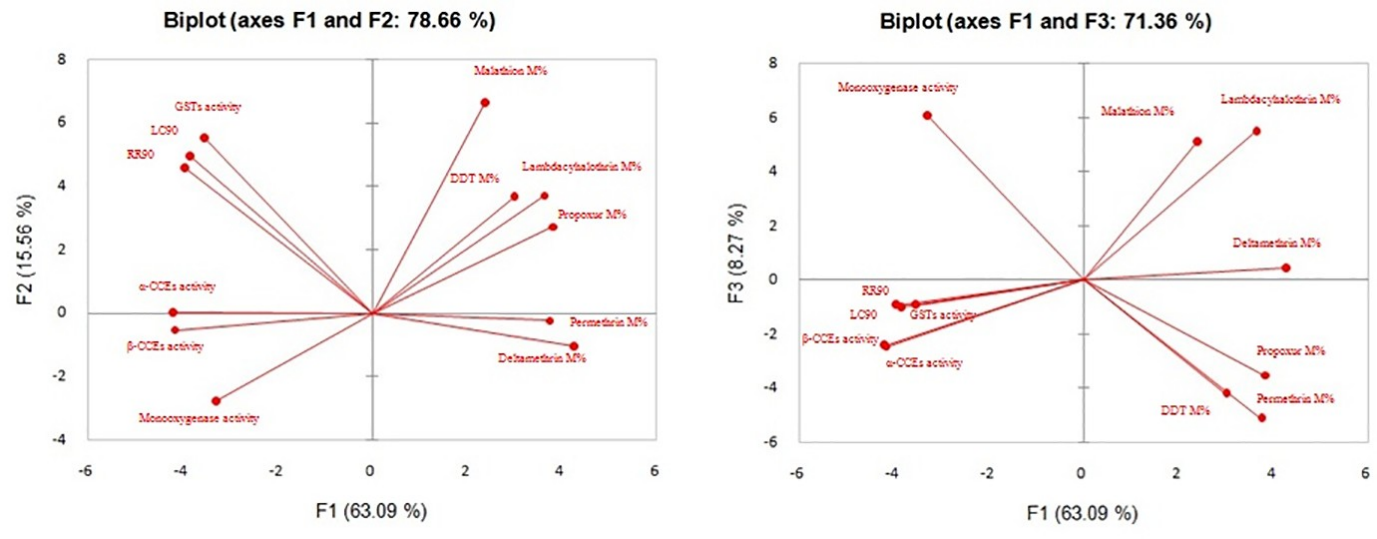
Biplot axis showing the association of different studied variables and insecticide resistance in *Culex quinquefasciatus* through principal component analysis.

Principal Component Analysis was performed using 12 different variables *i*.*e;* (i) LC90 and RR90 values of *Culex quinquefasciatus* populations against WHO recommended dose of temephos (ii) mortality percentage of adult mosquitoes against deltamethrin, lambdacyhalothrin, permethrin, malathion, propoxur and DDT and (iii) quantitative activity of major detoxifying enzymes (Fig 6). The 1^st^ PCA axis represented 63.09% of the information while the 2^nd^ and 3^rd^ axis represented 15.56% and 8.27% respectively. The 1^st^ and 2^nd^ axis together explained 78.66% of the information about the study. LC90 and RR90 values against temephos in the larval population of *Cx*. *quinquefasciatus* showed significant positive correlation with the elevated level of GSTs activity [correlation (r) = 0.97, p = 0.05]. Likewise, positive correlation was also observed between larval resistance and the elevated levels of α-CCEs (r = 0.71, p = 0.05) and β-CCEs (r = 0.76, p = 0.05). However, no significant correlation was found between the larval resistance and adult resistance against the insecticides used in the study. Mortality percentages of three synthetic pyrethroids were positively correlated with one another [deltamethrin and permethrin (r = 0.83, p = 0.05), deltamethrin and lambdacyhalothrin (r = 0.74, p = 0.05), lambdacyhalothrin and permethrin (r = 0.50, p = 0.05)] thereby showing the development of resistance against synthetic pyrethroids. Moreover, the quantitative activity levels of α-carboxylesterases and β-carboxylesterases did not correlate with resistance to synthetic pyrethroids. Resistance to DDT and propoxur was also found to be positively correlated (r = 0.72, p = 0.05) indicating multiple resistance to different groups of insecticides.

## Discussions

Ever since the first report of resistance in the mosquito vectors in 1950s, research works have been carried on throughout the world to elucidate and clarify the mechanisms behind insecticide resistance development. Research unveiling the resistance mechanism holds importance by directing towards new techniques and strategies of insecticide application against the mosquito vectors. The current study reports insecticide susceptibility/resistance status of the vector *Culex quinquefasciatus* against four different classes of insecticides *i*.*e*., organophosphate (temephos and malathion), carbamate (propoxur), synthetic pyrethroids (deltamethrin, lambdacyhalothrin and permethrin) and an organochlorine (DDT) from the highly populated areas of three districts of Northern part of West Bengal, India.

The susceptibility assays of *Cx*. *quinquefasciatus* larvae against temephos shows that the field populations have developed resistance against this organophosphate evident by RR_90_ values of the tested samples. Out of the eight field population under study, JPT with RR_90_ = 1.33 was found to be the sole temephos susceptible population while CPR showed a sign of mild resistance (RR_90_ = 2). With RR_90_ values 18 and 11.33, SHM and ISL populations showed severe resistance to temephos. Similar resistance to another organophosphate used in the study (malathion) was assessed in *Cx*. *quinquefasciatus* adults with mortality rate found to be as low as 3.22% in ISL population. This observed resistance against malathion in all of the tested populations (Table 2) may be due to the exposure of mosquitoes to malathion from agricultural runoff. The three districts of West Bengal under study namely Darjeeling, Jalpaiguri and Uttar Dinajpur rely on the agricultural sector for a huge part of their economy. Moreover, these districts are heavily dependent on tea plantation practices which are widely spread among the entire region and also partly on pineapple cultivation (Uttar Dinajpur) and areas in Jalpaiguri district upon jute cultivation. The insecticide malathion used in huge amount for the agricultural purposes might be the reason behind resistance development of *Cx*. *quinquefasciatus* against it owing to the close vicinity of human dwellings and residential areas and the tea plantation areas and other agricultural fields [21]. The contamination of breeding habitat of *Cx*. *quinquefasciatus* by the accumulation of malathion used in agricultural sector leads to indirect exposure of mosquitoes and in turn to the development of resistance against the same. Similar findings of resistance development to agriculturally sprayed malathion in *Aedes aegypti* population of this region has already been reported by Bharati and Saha (2018) [22]. Furthermore, there are similar reports on the development of resistance by field populations of *Cx*. *quinquefasciatus* vectors owing to the indirect exposure of malathion used primarily for agricultural practices in India [23] and worldwide [24].

Moderate to high level of resistance towards temephos found in the *Cx*. *quinquefasciatus* larvae may be related to the regular use of this organophosphate for dengue and malarial vector control both by the Government and other vector management agencies. This resistance reported is of concern because temephos being easily available in the market and budget friendly is one of the most widely used larvicide in India [7] mainly for the control of dengue and malaria. Application of temephos in the drains and stagnant water bodies in and around the residential areas in addition to the targeted dengue and malarial vectors, also indirectly targets the filarial vector *Cx*. *quinquefasciatus*. This non targeted exposure of the vector to temephos might be the reason behind temephos resistance found in the current study from three districts of West Bengal which being dengue endemic region has regular and heavy spray of temephos in order to subside the disease vectors.

In mosquitoes and other insect species, elevated level and over expression of non specific carboxylesterases is associated with the development of resistance to organophosphate insecticides [11]. Therefore, in the present study, highest quantitative value of α-CCEs and β-CCEs activity found in SHM and ISL population as compared to the other tested populations might be linked to higher level of temephos resistance in SHM and ISL. Likewise, low mortality rates suggesting severe resistance to malathion in ISL and FLB may also be coupled with enhanced activity of both α-CCEs and β-CCEs of these populations among all other tested populations (Tables 4 and 5). Associations of similar kind in organophosphate resistant mosquitoes to elevated levels of non specific CCEs have been reported around the globe [22, 25–26]. Amplification of non specific esterases is the most frequently observed resistance mechanism in organophosphate resistant populations of *Culex* and *Aedes*[27].

Moreover, the different number of isozymes in the qualitative study through NATIVE PAGE should also be reflected upon. In this study, SHM, ISL, FLB and CPR was found to express four different bands for α-CCEs thereby expressing higher number of isozymes thought to be associated with resistance development against organophosphates (Fig 4) (Table 4). On contrary, JPT expressed only two bands for α-CCEs and three bands for β-CCEs which were not as deeply stained as those of SHM and ISL (Figs 4 and 5). Presence of darkly stained bands in SHM and ISL may correspond to the over expression of that particular α-CCEs isozyme. Similar association of organophosphate resistance with elevated level of esterases in field population of *Cx*. *quinquefasciatus* was studied and reported by Pietrantonio *et al*., 2000 [26]. The expression of four different bands for α-CCEs and one deeply stained band for β-CCEs in CPR population in spite of its very slight resistance to temephos might be suggestive of the role of non specific esterases in resistance development towards malathion as adult *Cx*. *quinquefasciatus* of CPR expressed low mortality rate when assayed against malathion (Table 2). Furthermore, higher number of bands for α-CCEs and β-CCEs in FLB and BDN (Tables 4 and 5) might also be taken to be associated with malathion resistance to some extent. However, higher number and also the greater intensity of bands for both α-CCEs and β-CCEs of all the studied field populations of *Cx*. *quinquefasciatus* when compared to that of SP might itself impart light upon the association of elevated levels of CCEs with resistance of mosquitoes to organophosphate insecticides [11]

Overproduction of a major detoxifying enzyme group GSTs was reported to be associated with resistance development in mosquito vectors to organophosphates [28]. Maximum of 80 fold higher quantitative values of GSTs calculated in the present study, on comparison to GSTs activity level of SP might be considered for its association with resistance development to organophosphates among the tested populations. Higher values of GSTs activity in SHM and ISL similar to the elevated esterase value of the same suggest the role of this enzyme in the onset of resistance.

Bioassay tests in the current study of field populations against propoxur—a carbamate insecticide showed severe resistance to propoxur with mortality percentage below 63 for all of the tested populations (Table 2). There are similar reports of resistance to propoxur in *Cx*. *quinquefasciatus* from different parts of the world [29–30]. In India, till date, propoxur has not been used as a mosquitocidal tool [7], however, severe resistance to propoxur found in this study may be due to indirect exposure to other propoxur-containing insect repellents used domestically. In addition, *Cx*. *quinquefasciatus* being indoor mosquito is prone to untargeted exposure to such repellents used in households thereby developing secondary resistance.

Resistance to carbamates in mosquitoes and other arthropod species has generally been associated with increased level of CCEs activity [11] and seldom through CYP_450_s and GSTs [31]. Since, the quantitative levels of detoxifying enzymes studied varied among the tested population, on contrary to a similar mortality rate against propoxur in the population examined, no correlation could be established between the observed pattern of propoxur resistance and overproduction of metabolic enzymes.

The present study shows resistance to three synthetic pyrethroids used (deltamethrin, lambdacyhalothrin and permethrin) by field populations of *Cx*. *quinquefasciatus*. JPT population with mortality rate of 95.65% lies in the intermediate / incipient resistance state to permethrin, rest of the populations have developed resistance to all three synthetic pyrethroids tested. Similar results encasing pyrethroid resistance in *Cx*. *quinquefasciatus* have been reported worldwide [32–35]. Resistance to pyrethroids in *Cx*. *quinquefasciatus* from three districts of West Bengal and also from around the globe is of major concern since pyrethroids are the sole insecticides used in LLITNs. Reports on low efficacy of pyrethroid impregnated bed nets against *Cx*. *quinquefasciatus* have already come to limelight [36–37] and this could hamper the success of vector control programs in near future. The observed resistance to pyrethroids may be the result of selection mainly from the domestic use of mosquito repellents, coils, oils and sprays that usually contain pyrethroids. *Cx*. *quinquefasciatus* being anthropophilic and indoor resting mosquitoes are more likely to get exposed to these household mosquito repellents than any other mosquito species. Furthermore, application of synthetic pyrethroids in the agricultural sector may also be involved in resistance development [38], as the residue collects and accumulates in the adjoining drains–the most preferred habitat of *Cx*. *quinquefasciatus* thereby building a selection pressure in the vector.

CYP_450_ family of metabolic enzymes is involved and found to be associated with pyrethroid resistance in mosquito vectors [39]. In this study, elevated CYP_450_ level might thereafter indicate a possibility of CYP_450_ enzyme group to be involved in pyrethroid resistance. The higher quantitative values of CYP_450_s in SHM, FLB, BDN and DPG (Table 3) do coincide with the lower mortality rates of these populations against three pyrethroids used (Fig 2). Studies on the involvement of CYP_450_ enzyme group in pyrethroid resistance in *Cx*. *quinquefasciatus* and also the different type of CYP_450_ involved confers positive correlation between resistance and increased enzyme activity [9]. Quantitative esterase activity could not be linked with pyrethroid resistance in the present study thereby suggesting inefficacy of *Culex* esterases against pyrethroids [40]. Similarly no correlation between elevated esterase level and pyrethroid resistance was observed in *Cx*. *quinquefasciatus* from USA [41].

Apart from the detoxifying enzymes, another mechanism underlying pyrethroid resistance is target-site insensitivity of the voltage-gated sodium channel [11]. Mutation in the voltage-gated sodium channel gene termed as knock down resistance (kdr) mutation leads to the development of resistance to synthetic pyrethroids [11]. Hence, along with metabolic enzymes, studies on kdr mutation should also be equally emphasized upon. KDT_50_ and KDT_99_ values (>60 minutes) of the tested population against pyrethroids suggests lower efficiency of pyrethroids and prolonged exposure needed to control *Cx*. *quinquefasciatus* as study of KDT for the detection of resistance in WHO test tubes is a good indicator of resistance [42].

Severe resistance to an organochlorine (DDT) in the studied population may be associated with widespread use of this insecticide in the vector management programs [33]. Mortality rate as low as 3.03% (BDN) has been observed in the present study and similar DDT resistant populations have also been reported from various region worldwide [43]. Association of GSTs with DDT resistance could not be positively correlated thereupon imparting the thought on the involvement of kdr mutation in *Cx*. *quinquefasciatus* populations of the studied region. Comparatively higher KDT_50_ and KDT_99_ values of DDT than those of the synthetic pyrethroids from the study suggest the inefficiency of DDT as an insecticide in vector control. However, studies on synergist assay and presence of major kdr mutations might provide a clear insight into the mechanism conferring resistance.

## Conclusion

The present study provides the first ever report of varied level resistance in *Cx*. *quinquefasciatus* towards few insecticides belonging to four different classes from three districts of northern region of West Bengal. This multiple resistance in *Cx*. *quinquefasciatus* from the Northern part of West Bengal is usually a secondary resistance due to indirect exposure to insecticides. Coupling the over-expression of metabolic enzymes to resistance as reported in the present study might help in designing new vector management strategies in this region. Besides, the over-production of metabolic enzymes upon insecticide exposure should be further investigated for better characterization of their role in resistance development Therefore, regular surveillance of resistance status to different insecticides in the field collected populations of mosquito vectors is vital for proper management of vectors and in turn vector-borne diseases.

## Supporting information

S1 Table Sampling sites and their geographical coordinates. (DOCX)
