# Supplementary material for: Insecticide resistance mapping in the vector of lymphatic filariasis, Culex quinquefasciatus Say from northern region of West Bengal, India
Source: PLoS One. 2019 May 29;14(5):e0217706. doi: 10.1371/journal.pone.0217706 (PMC6541298; doi:10.1371/journal.pone.0217706)
Supplement: S1 Table — (DOCX) [file pone.0217706.s001.docx]

Table S1: Sampling sites and their geographical coordinates.

| **District** | **Site** | **Geographical coordinates** |
| --- | --- | --- |
| Darjeeling | Shivmandir | 26.71° N, 88.35° E |
|  | Siliguri | 26.73° N, 88.40° E |
|  | Bidhannagar | 26.49° N, 88.22° E |
| Jalpaiguri | Jalpaiguri town | 26.54° N, 88.72° E |
|  | Fulbari | 26.69° N, 88.45° E |
|  | Dhupguri | 26.58° N, 89.00° E |
| Uttar Dinajpur | Chopra | 26.37° N, 88.31° E |
|  | Islampur | 26.25° N, 88.19° E |
